# Master Settlement Agreement (MSA) Spending and Tobacco Control Efforts

**DOI:** 10.1371/journal.pone.0114706

**Published:** 2014-12-15

**Authors:** Jayani Jayawardhana, W. David Bradford, Walter Jones, Paul J. Nietert, Gerard Silvestri

**Affiliations:** 1 Department of Health Policy and Management, College of Public Health, University of Georgia, Athens, Georgia, United States of America; 2 Department of Public Administration and Policy, School of Public & International Affairs, University of Georgia, Athens, Georgia, United States of America; 3 Health Administration & Policy, Medical University of South Carolina, Charleston, South Carolina, United States of America; 4 Department of Medicine, Medical University of South Carolina, Charleston, South Carolina, United States of America; University of Alabama, United States of America

## Abstract

We investigate whether the distributions to the states from the Tobacco Master Settlement Agreement (MSA) in 1998 is associated with stronger tobacco control efforts. We use state level data from 50 states and the District of Columbia from four time periods post MSA (1999, 2002, 2004, and 2006) for the analysis. Using fixed effect regression models, we estimate the relationship between MSA disbursements and a new aggregate measure of strength of state tobacco control known as the Strength of Tobacco Control (SoTC) Index. Results show an increase of $1 in the annual per capita MSA disbursement to a state is associated with a decrease of −0.316 in the SoTC mean value, indicating higher MSA payments were associated with weaker tobacco control measures within states. In order to achieve the initial objectives of the MSA payments, policy makers should focus on utilizing MSA payments strictly on tobacco control activities across states.

## Introduction

Tobacco use has been a major public health problem in the United States for many decades. Recent estimates find that tobacco consumption is the primary cause of 443,000 premature deaths in the U.S. each year while about 8.6 million people suffer from smoking related illnesses [1]. Associated tobacco-related average annual health care costs in the U.S. totaled approximately $96 billion in 2001–2004, not including second hand smoke exposure and productivity losses [1].

Despite these statistics tobacco manufacturers were not held liable for health care costs associated with tobacco consumption for many years. This changed in 1998 when the four largest tobacco companies in the U.S., as the original participating manufacturers (Brown & Williamson, Lorillard, Philip Morris, and R.J. Reynolds) entered into a settlement agreement with the Attorneys General of 46 states and the District of Columbia to help offset the excess burden on state Medicaid programs from tobacco-related diseases. This settlement, known as the Tobacco Master Settlement Agreement (MSA), involved a large financial transfer from cigarette manufacturers, both the original and subsequent participating manufacturers, to the states. The MSA was actually a much watered-down agreement from the one that had been negotiated two years earlier by the states but was then defeated by the U.S. Senate after escalating demands for money grew too much for the tobacco industry to accept [2]. The stated rationales for the payments were to help pay for tobacco-related health care costs and to fund public education and other tobacco control activities [3]. The MSA required payment of $206 billion to the states over 25 years, $1.5 billion over 10 years to support state anti-smoking measures, and $250 million to fund research into reducing youth smoking. Prior to the MSA, four other states (Florida, Minnesota, Texas, and Mississippi) entered into separate individual agreements with the tobacco industry whereby those states would receive settlements totaling $40 billion over the same 25-year period [4].

There was a general public assumption that MSA payments would be spent on anti-tobacco measures and would be used by states to implement policies to discourage tobacco (especially cigarette) consumption. However, under the terms of the MSA state spending was not legally constrained. Indeed, since MSA payments are lump-sum revenue transfers, states have been able to divert much of the MSA money towards other uses [5].

In this study, we systematically examine the link between MSA funding flow to each state and the degree to which state tobacco control efforts discourages tobacco consumption. We estimate these associations by constructing a panel data set containing MSA fund disbursements, state socioeconomic characteristics, and an aggregate measure of the state tobacco control environment, known as the Strength of Tobacco Control (SoTC) index [6]. Using a fixed effects model that controls for state specific characteristics, we find that greater levels of MSA disbursements are associated with less stringent tobacco control environments.

Critics of the MSA implementation have long pointed out that most states have not directed MSA funds toward tobacco control among youth or treatment of tobacco-related diseases. A study by the General Accounting Office in 2000–2001 concluded that “states have used their MSA payments for a variety of programs and budget priorities, including, but not limited to, tobacco control and health care programs” [7]. The study suggested that although the largest portion of the MSA funds have been allocated to state health care programs, they have not necessarily focused on youth antismoking programs or tobacco related health care costs.

Between 2001 and 2006 a number of similar studies provided evidence that much MSA funding was being used in ways that were inconsistent with the intentions of the agreement. According to the *Campaign for Tobacco Free Kids*, only five states spent MSA payments on youth anti-smoking programs at the 20–25% level recommended by the Centers for Disease Control and Prevention (CDC). Indeed, Michigan, North Carolina, and Tennessee did not spend any MSA money on anti-tobacco activities in the early years [8]. The literature is unclear on the relationship between tobacco control funding and state tobacco control activities. Much of the work to date on the association between funding and tobacco control has been cross-sectional in nature. While a few studies have examined variations across time for individual states, no study has analyzed how all states in the U.S. have fared in terms of usage of MSA funding over time.

Nevertheless, the associations identified to date are informative. Some studies have been able to suggest possible state-specific factors related to tobacco policymaking. For example, Austin-Lane, Girasek and Barbour used a modified Delphi process with tobacco policy professionals [9]. They found that most important influences on state tobacco control activities were budgetary constraints, lobbying, advocacy, tobacco economy, legislative priorities, public opinion, and leadership by the governor or state legislators. In recent years, several econometric efforts have been made to examine the relationship between MSA funding and state tobacco control activities. Sloan and his co-authors examined the determinants of state MSA funds allocation, and found that tobacco-producing states and states with high percentages of conservative Democrats, seniors, African-Americans, Hispanics, or high-income individuals tended to spend less on tobacco control [10]. In contrast, states with strong education and health care lobbies were more likely to have high rates of spending on tobacco control.

Perhaps the most detailed effort to relate state tobacco policymaking to health outputs and outcomes has been conducted by Stillman and co-authors in their evaluation of the American Stop Smoking Intervention Study (ASSIST), conducted as collaboration between the National Cancer Institute (NCI) and 17 states between 1991 and 1999 [6]. ASSIST was an NCI demonstration project that existed prior to the establishment of the MSA. Through ASSIST, the NCI provided funding and helped states change social, cultural, economic and environmental factors that influence smoking through actual policy development and implementation. Tobacco-control policies included promotion of smoke-free workplaces, countering tobacco advertising and promotion, limiting tobacco access, and increasing tobacco prices through taxation. The ASSIST program and its funding ended in September 1999, only eight months after the MSA was enacted. However, the ASSIST research did develop a validated measure of state tobacco control environment, the *Strength of Tobacco Control Index*. Even though the ASSIST program ended soon after the MSA took effect, the SoTC Index has been continuously updated, and has been measured approximately every two years (in 1999, 2002, 2004 and 2006) across all states. It should be noted that ASSIST program was implemented in three phases where the first two years were devoted to planning and coalition building, the next four years devoted to intervention and implementation phase, and the last two years devoted to evaluation. It should be noted that the description of the ASSIST program in this manuscript may not include a full description. Please refer to National Cancer Institute (2006) for more details of the ASSIST program.

The SoTC index was developed by ASSIST program researchers to evaluate whether states with better resources and infrastructure and with an improved capacity to deliver tobacco control programs will have better success in achieving lower levels of tobacco consumption and smoking prevalence [11]. The SoTC has been adjusted for state-specific differences in resources, capacity, and efforts toward tobacco control, and has been validated as a measure of tobacco control intensity at the state level [6].

Some research studies have used SoTC index to investigate its impact on smoking prevalence rates. Using data from Tobacco Use Supplement to the Current Population Survey, Jemal et al. (2011) examined the impact of SoTC on smoking prevalence among both men and women from 1992–93 to 2006–07 and found the decline in smoking prevalence rates among women to be associated with SoTC index but not among men [12]. York et al. (2010) used county level data from a randomized controlled trial to examine associations between SoTC scores and smoking prevalence and found no correlation between the two variables [13].

## Methods

We investigate whether MSA spending is associated with stronger (or weaker) tobacco control, using the SoTC Index and its three sub-components: resources, capacity and efforts. We assume that state policy makers make decisions in an attempt to satisfy state residents' (e.g., the median voter's) preferences – which leads to competing interests. Thus, inflows of revenues may be divided across multiple policy goals, and not necessarily allocated fully to the area for which the funds have been intended. Since MSA funds are not technically earmarked, they are fungible and may subsidize many state programs beyond tobacco control. To study the extent of “leakage” from tobacco control, we looked to see if there were any significant relationships between per capita MSA payments and states' SoTC and subcomponent scores.

### Data

We combined multiple data sources to create a panel data set for the analysis. The SoTC Index and its component data (for 1999, 2002, 2004, and 2006) are maintained by the Health Policy Center in the Institute for Health Research and Policy at University of Illinois at Chicago [14]. Data on MSA disbursement to each state and details about whether MSA payments were securitized are from the National Conference of State Legislatures [15]. The MSA spending data includes information on various categories of spending such as education, health, and tobacco prevention activities, and the amount of the securitization of MSA payments at the state level. We also captured the percentage of MSA money that was allocated towards tobacco prevention activities by each state [15].

Data on state tobacco excise taxes are from the State Cancer Legislative Database of the National Cancer Institute [16]. Per capita income data are from the Bureau of Economic Analysis at the U.S. Department of Commerce while population and other state socioeconomic data are from the U.S. Census Bureau. Data on physician populations were extracted from publications of the American Medical Association. Our final data sample consists of data on 50 states and the District of Columbia for 4 years (N = 204). Table 1 shows the descriptive statistics of the data.

**Table 1:** Summary Statistics of the Variables.

Variable	Mean	Standard Deviation
SoTC overall index	0.03	0.91
Effort index component of SoTC	−0.22	1.16
Resources index component of SoTC	0.29	0.89
Capacity index component of SoTC	−0.04	0.99
MSA Payments to state, per capita	$24.33	$13.28
Indicator variable = 1 after state securitizes any MSA payment	0.24	0.43
Amount of MSA payment securitized in year, per capita	$23.71	$86.10
Excise taxes on cigarettes, per pack	$0.70	$0.54
State per capita income	$31,209.33	$6,004.56
State population under age 18 (1000 s)	1,422.10	1,651.81
State population aged 19 to 24 (1000 s)	554.18	627.20
State population aged 25 to 64 (1000 s)	2,976.75	3,320.89
State population over age 65 (1000 s)	704.39	761.45
Percent of state population graduated high school	85.90%	4.00%
Number of physicians per 1000 population	2.93	1.01
Percent of state population that is Black	11.30%	11.60%
Percent of state population that is Hispanic	8.32%	9.13%
Percent of state population that is other race (Non-White)	80.40%	14.00%

Note: Total number of observations is 204. Source: Authors' analysis of the dataset used in the study. Please see ‘data’ section for details.

#### Dependent Variables

*The Strength of Tobacco Control Index:* The SoTC Index is made up of three constructs: resources allocated to tobacco control activities within states, capacity for state-level tobacco control, and program efforts focused on tobacco control policy and social-environmental change [6]. These constructs are assembled using 41 separate measures of the state policy environment [6]. The “resources” construct measures the amount of money received by the state health department and major agencies in the state, and the number of full time staff assigned to tobacco control activities in the state. Since the link between general fund allocation and State Department funding in states is weak according to the literature, it is very unlikely that MSA funds may be included in State Department funding, thus minimizing possible endogeneity bias. The “capacity” construct measures the potential ability of each state in performing tobacco control activities given the resources, including such variables as state leadership support for tobacco control and the relationships between and experience held by health programs in tobacco control activities [6]. The “efforts” construct measures the amount of tobacco control activities that each state is engaged in, such as policy, media and program services. Finally, these three constructs are aggregated into one over-arching index – the SoTC – which captures the overall tobacco control environment in each state at each point in time [6]. The SoTC does not actually measure smoking prevalence and intensity in the state. Rather, it is a measure of tobacco control efforts that states undertake to reduce tobacco consumption in each state.

Higher values of the index indicate stronger attempts to control tobacco. By construction, this index has a mean of approximately zero, and ranges from around −2 to around +4 (though slightly higher values are possible and observed in the data). Annual state-level measure of this index will serve as our key dependent variable in the analysis.

#### Independent Variables

*MSA Payment:* There are two considerations for how we include MSA payments in the models. First, these annual payments vary by the number of smokers and estimated tobacco-related Medicaid expenditures in each state [2]. Consequently, we measure the impact of annual MSA payments as MSA dollars disbursed per smoker. Second, to control for possible non-linearities in state responses, we include MSA spending per smoker squared in the analysis. We also carried out the regressions using per capita MSA payments and its square in the place of per smoker MSA payments, and the results were quite similar. Hence, we present only the results with MSA per capita in the paper.

*Securitization:* Securitization, allows states to essentially borrow up to the present value of the entire remaining stream of MSA payments (minus interest payments) in any year and pay the bonds back using the future MSA payments. Thus, a state can, and many have, consume its entire MSA benefit in one year. We capture the impact of securitization in two ways. First, we created an indicator variable which equals 1 in each year on or after a state securitizes any of the MSA funds (and is equal to zero otherwise). This variable measures whether states that front-load consumption of any amount of the MSA funds are different from states that do not; one might expect that states find it politically easier to transfer MSA funds to non-tobacco control uses, such as road construction, when they have been converted to lump sums, rather than coming in as scheduled payments with at least nominally predefined uses. Our second securitization variable is a measure of the per capita amounts of money taken each year as a result of bond issuance.

*Other state characteristics:* We also control for time-varying factors that might confound the relationship between MSA payments and state tobacco control efforts. We capture price effects on cigarette demand using state cigarette excise taxes per pack. We also explored using per pack cigarette prices, with results that were qualitatively similar; we use taxes because the literature is relatively evenly divided on which to use and treating excise taxes as exogenous variable, and excise taxes are less subject to measurement error [17]. Demand for cigarettes is also known to vary by demographic characteristics, so we include measures of per capita income, percent of the state's population with at least of high school diploma, population by age categories, and population by race and gender (in percentages). Since having access to healthcare could also affect tobacco use, we include the number of physicians per thousand people in the state. Finally, we include state-level fixed effects in order to capture time-invariant factors that affect cigarette demand and the state policy environment (e.g., if the state is a tobacco producing state).

### Analysis

The analyses of the data were performed using Stata version 11 (StataCorp LP, College Station, TX). We estimated 4 fixed-effect regression models using the SoTC index and its 3 sub-component constructs as dependent variables and other measures discussed above as independent variables.

It is important to note, however, that a state's spending on tobacco control programs could come from various sources such as income and property taxes, tobacco and alcohol taxes and MSA spending. In this analysis, we wish to identify the direct effect of MSA spending on tobacco control. Therefore, we exclude states' other spending on anti-tobacco activities from the analysis, which could also be endogenous with the SoTC measure.

We estimated the models as linear, log-transformed (after re-centering the SoTC index variables at 5), and as exponential transforms. The latter functional form performed best in terms of overall goodness of fit. Therefore, those results are presented in this paper. We also conducted our regression analyses excluding the states that securitized the MSA payments before 2006, and the results were quite similar to the results we obtained when we included all states in the analyses. Please refer to the supporting information file – S1 Analysis for a detailed description of the fixed effects model used in the analysis.

## Results

**Figure 1:**
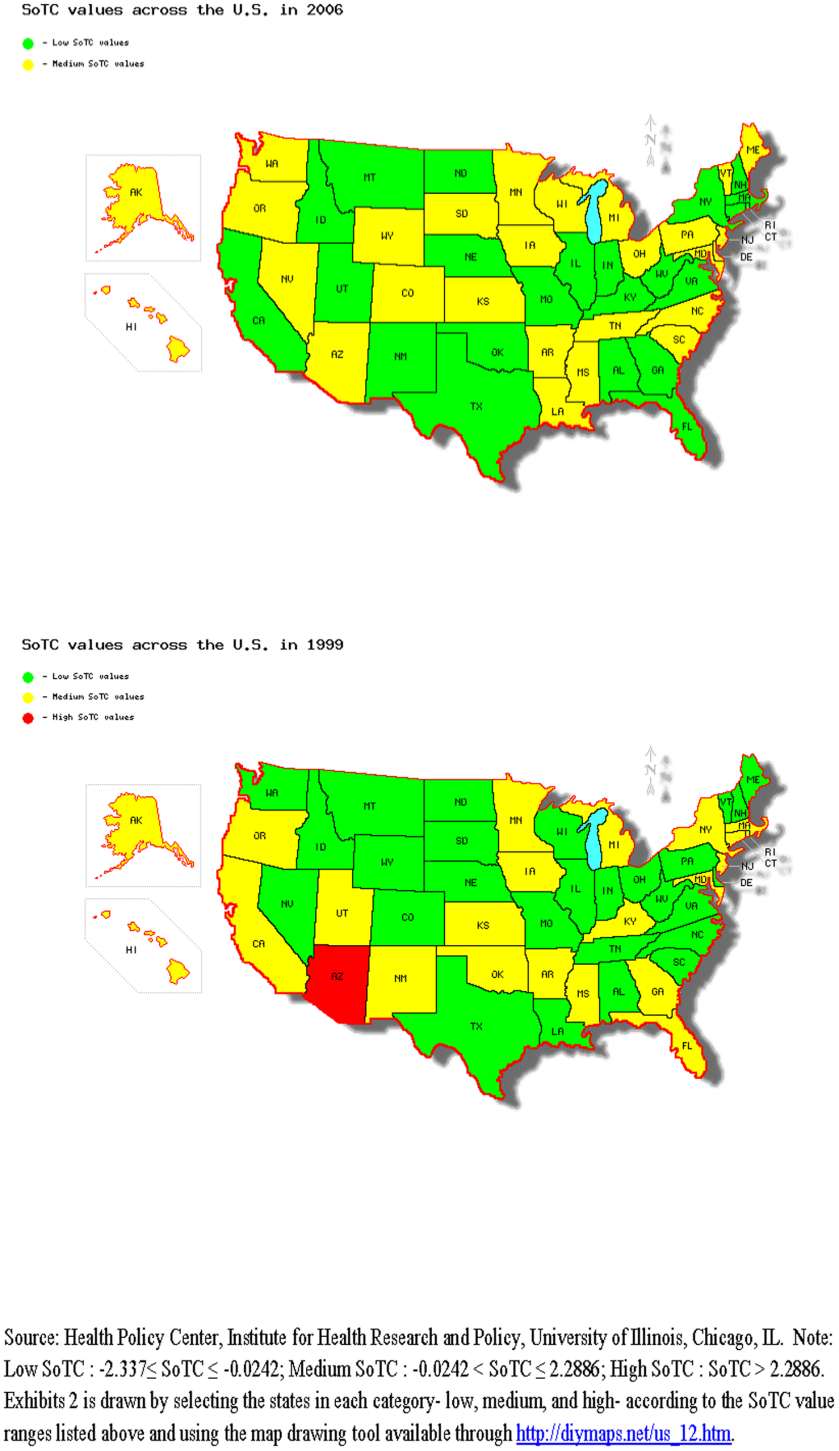
SoTC Values across the U.S. in 2006 and 1999.

Fig. 1 shows how each state in the U.S. has fared in terms of SoTC values in 1999 and in 2006, the starting and ending years of the time period in this study. After ranking the SoTC values from lowest to highest for year 1999, we divided the dataset in to three equal-sized groups by SoTC values – low, medium and high. Fourteen states (ex. LA, SD, NC, etc) improved their SoTC values from 1999 to 2006 (moving from low to medium) while other states fell short of improving SoTC values. For example, while Arizona ranked as a state with high SoTC value in 1999, its SoTC value dropped to medium rank by 2006. Similarly, SoTC values in twelve other states (ex. MI, FL, GA, etc.) also dropped from 1999 to 2006 indicating poorer tobacco controlling activities.

The reasons for some states to improve their SoTC values while others experience a decrease in their SoTC values could lie behind how each state spent their MSA payments. For example according to Sloan et al (2005), North Carolina - a tobacco producing state, allocated more than half of the initial MSA funds towards tobacco growers and communities negatively affected by the MSA. In 1999, American Cancer Society and American Lung and Heart Association requested 10% of the state MSA funds be allocated to tobacco control. However, in 2002, a North Carolina healthcare organization that subsidized senior's prescription drugs purchases also received some NC MSA money. In 2003, $65 million of NC MSA funds were diverted to reduce the state's budget deficit; at the same time the state allocated $6.2 million from the Health and Wellness Trust fund to sponsor a teen-smoking prevention campaign for up to three years, while another $3 million was allocated to children's obesity prevention program [5]. The SoTC values of NC varied from −0.272 in 1999 to −1.101 in 2002 to 0.633 in 2006. Thus, the initial diversion of MSA funds to non-tobacco related activities appears to be reflected in the SoTC, as was the subsequent increase in the allocations of MSA funds in later years towards tobacco control activities.

On the other hand, Michigan, a non-tobacco producing state, allocated 75% of their MSA funds toward the Michigan Merit Award Program, a scholarship program that provides funding for high school students who do well on a proficiency exam while the other 25% was allocated to a Tobacco Settlement Trust Fund that funds various health programs including a prescription drug program for seniors and non-tobacco related research efforts [5], [18]. These early MSA fund allocations, mostly away from tobacco control activities in the state, help explain the downward trend in SoTC values of Michigan from 0.492 in 1999 to 0.038 in 2006.

Table 2 shows the average SoTC values across the four time periods for each state starting from highest value to the lowest value. Alaska, Arizona, Mississippi, New Jersey, and New Mexico have the highest Average SoTC values; the District of Columbia, Tennessee, Ohio, Louisiana, and North Dakota have the lowest average SoTC values.

**Table 2:** Average SoTC values across the 4 years (99, 02, 04, 06) by state, from highest to lowest.

State	Average SoTC by state
Alaska	1.282
Arizona	1.229
Mississippi	1.023
New Jersey	0.882
New Mexico	0.801
Arkansas	0.756
Iowa	0.619
Maine	0.603
Maryland	0.541
Rhode Island	0.465
Hawaii	0.455
Oregon	0.448
West Virginia	0.389
Wyoming	0.387
California	0.375
Kansas	0.357
Minnesota	0.331
Kentucky	0.259
Florida	0.223
Colorado	0.195
Washington	0.155
Delaware	0.146
Georgia	0.138
New York	0.128
Nevada	0.123
Massachusetts	0.108
Michigan	0.065
Vermont	0.018
South Dakota	−0.028
Wisconsin	−0.116
New Hampshire	−0.161
Idaho	−0.168
Pennsylvania	−0.206
Connecticut	−0.228
North Carolina	−0.235
South Carolina	−0.245
Texas	−0.345
Oklahoma	−0.367
Missouri	−0.421
Alabama	−0.49
Utah	−0.509
Virginia	−0.515
Illinois	−0.589
Montana	−0.631
Nebraska	−0.68
Indiana	−0.734
North Dakota	−0.743
Louisiana	−0.79
Ohio	−0.821
Tennessee	−0.94
District of Columbia	−1.238

Source: Authors' analysis of the data set used in the study.

Results from the fixed effects regression models of tobacco control policy including all states in the analyses are presented in Table 3 while Table 4 presents regression results when excluding the states that securitized the MSA payments before 2006. Since results in Table 4 are quite similar to results in Table 3, we only discuss results of Table 3 in here. The first column includes the parameters from the overall SoTC model; columns 2–4 present parameters from the three sub-component models, and t-statistics are shown in the parentheses. Per capita MSA payments have a negative and statistically significant effect on the overall exponentiated SoTC policy variable. When calculated at the average of the SoTC data, the net marginal effect of MSA payments on overall SoTC is −0.316, which reflects how a $1 change in the annual per capita MSA disbursement would affect SoTC. In other words, a 10% increase in the annual per capita MSA disbursement (which is around $2.43) would be associated with a decrease of −0.76 in a state's SoTC value. Thus, we find that higher MSA payments are associated with weaker tobacco control environments.

**Table 3:** Fixed effects regression results of SoTC index and sub-component indexes.

Variable	Exp(SoTC overall index)	Exp(Resources index component of SoTC)	Exp(Capacity index component of SoTC)	Exp(Effort index component of SoTC)
MSA payments to state, per capita	−0.543	−4.877	−0.004	0.082
	(2.61)**	(2.92)**	(0.12)	(0.52)
MSA payments to state, per capita, squared	0.005	0.041	0.00	−0.001
	(2.11)*	(2.38)*	(−0.62)	(−0.62)
Indicator variable = 1 after state securitizes any MSA payment	2.498	26.461	−0.266	−1.879
	(−1.03)	(−1.36)	(−0.67)	(−1.03)
Amount of MSA payment securitized in year, per capita	−0.002	−0.034	0.00	0.002
	(−0.29)	(−0.57)	(−0.14)	(−0.31)
Excise taxes on cigarettes, per pack	−2.545	−22.891	0.198	−1.52
	(−1.18)	(−1.32)	(−0.56)	(−0.93)
State per capita income	0.00	0.002	0.00	0.001
	(−0.44)	(−0.37)	(−0.29)	(−1.72)
State population under age 18 (1000 s)	−0.013	−0.069	−0.004	−0.008
	(−0.68)	(−0.46)	(−1.31)	(−0.55)
State population aged 19 to 24 (1000 s)	−0.014	−0.047	−0.003	−0.043
	(−0.46)	(−0.19)	(−0.56)	(−1.82)
State population aged 25 to 64 (1000 s)	0.021	0.173	0.001	0.011
	(−1.49)	(−1.52)	(−0.34)	(−1.00)
State population over age 65 (1000 s)	−0.113	−0.991	0.005	−0.011
	(2.23)*	(2.44)*	(−0.61)	(−0.29)
Percent of state population graduated high school	−0.332	−1.585	0.045	−0.65
	(−0.67)	(−0.39)	(−0.55)	(−1.72)
Number of physicians per 1000 population	−1.173	−8.298	0.708	−2.448
	(−0.33)	(−0.29)	(−1.19)	(−0.9)
Percent of state population that is Black	−21.615	−473.351	−11.859	157.238
	(−0.17)	(−0.47)	(−0.58)	(−1.67)
Percent of state population that is Hispanic	−25.949	−512.454	−12.35	158.719
	(−0.21)	(−0.51)	(−0.6)	(−1.69)
Percent of state population that is other race (Non-White)	−22.838	−485.515	−12.059	158.205
	(−0.18)	(−0.48)	(−0.59)	(−1.68)
Percent of state population that is Female	2.205	24.538	0.497	−8.016
	(−0.52)	(−0.73)	(−0.72)	(2.52)*
Percent of state population that is Male	−0.979	−14.622	−0.892	9.161
	(−0.22)	(−0.4)	(−1.19)	(2.68)**
Year = 2002	9.674	82.779	−0.176	−2.468
	(2.17)*	(2.31)*	(−0.24)	(−0.73)
Year = 2004	10.704	91.712	0.11	−4.431
	(−1.92)	(2.05)*	(−0.12)	(−1.05)
Year = 2006	12.632	108.042	−0.553	−5.012
	(−1.55)	(−1.65)	(−0.41)	(−0.82)
Constant	2,301.85	48,545.89	1,222.75	−15,806.27
	(−0.18)	(−0.48)	(−0.6)	(−1.68)
*R* ^2^	0.24	0.25	0.17	0.19
*Observations*	204	204	204	204

Source: Authors' analysis of the data set used in the study.

Note: T-statistics are shown in the parentheses.

*p<0.05;

**p<0.01.

**Table 4:** Fixed effects regression results of SoTC index and sub-component indexes, excluding states that securitized MSA payments before 2006.

Variable	Exp(SoTC overall index)	Exp(Resources index component of SoTC)	Exp(Capacity index component of SoTC)	Exp(Effort index component of SoTC)
MSA payments to state, per capita	−0.741	−6.753	0.01	0.138
	(2.67)**	(3.03)**	(−0.24)	(−0.67)
MSA payments to state, per capita, squared	0.006	0.056	0.00	−0.002
	(2.20)*	(2.51)*	(−0.26)	(−0.76)
Excise taxes on cigarettes, per pack	−6.1	−53.323	0.322	−0.563
	(−1.8)	(−1.95)	(−0.62)	(−0.22)
State per capita income	0.00	0.001	0.00	0.001
	(−0.32)	(−0.11)	(−0.15)	(2.31)*
State population under age 18 (1000 s)	−0.004	−0.035	−0.007	0.013
	(−0.13)	(−0.16)	(−1.56)	(−0.65)
State population aged 19 to 24 (1000 s)	−0.045	−0.23	−0.005	−0.071
	(−0.74)	(−0.47)	(−0.55)	(−1.58)
State population aged 25 to 64 (1000 s)	0.042	0.31	0.003	0.019
	(−1.77)	(−1.62)	(−0.8)	(−1.07)
State population over age 65 (1000 s)	−0.222	−1.7	0.003	−0.073
	(2.33)*	(2.23)*	(−0.2)	(−1.05)
Percent of state population graduated high school	−0.547	−3.157	−0.014	−0.76
	(−0.77)	(−0.55)	(−0.13)	(−1.45)
Number of physicians per 1000 population	−3.505	−34.754	0.834	−0.805
	(−0.43)	(−0.53)	(−0.66)	(−0.13)
Percent of state population that is Black	13.266	−220.571	−18.075	222.123
	(−0.08)	(−0.16)	(−0.7)	(−1.79)
Percent of state population that is Hispanic	7.272	−270.704	−18.493	221.705
	(−0.04)	(−0.2)	(−0.71)	(−1.79)
Percent of state population that is other race (Non-White)	11.606	−233.36	−18.204	221.178
	(−0.07)	(−0.17)	(−0.7)	(−1.78)
Percent of state population that is Female	7.085	66.99	0.759	−9.916
	(−1.13)	(−1.33)	(−0.78)	(2.14)*
Percent of state population that is Male	−5.034	−50.15	−1.164	10.763
	(−0.78)	(−0.97)	(−1.17)	(2.26)*
Year = 2002	16.375	146.456	−0.387	−5.852
	(2.59)*	(2.88)**	(−0.4)	(−1.25)
Year = 2004	19.538	176.357	−0.033	−9.004
	(2.37)*	(2.66)**	(−0.03)	(−1.48)
Year = 2006	23.788	220.275	−0.946	−13.429
	(−1.86)	(2.15)*	(−0.48)	(−1.43)
Constant	−1,145.95	23,365.16	1,842.06	−22,115.30
	(−0.07)	(−0.17)	(−0.71)	(−1.78)
*R* ^2^	0.33	0.35	0.16	0.21
*Observations*	140	140	140	140

Source: Authors' analysis of the data set used in the study.

Note: T-statistics are shown in the parentheses.

*p<0.05;

**p<0.01.

One of the reasons for this finding might be that many MSA payments are not allocated towards tobacco control [8]. Note from columns 2–4 of Table 3 that this effect appears to be a result of the resources component of the SoTC index, where per capita MSA payments are statistically significant, though they are not significant in the regression results of the other two components. Taken together, these results are consistent with the concern that MSA payments have been diverted from tobacco control activities to other non-tobacco state priorities. For example, per capita MSA payments to the District of Columbia were very high ($54) in 1999 and further increased to $61 by 2006, while at the same time its SoTC value fell from −0.74 to −1.18. A similar pattern holds for Georgia (although Georgia MSA payments were not as high as for D.C.). These examples, illustrate that high per capita MSA payments are not necessarily associated with high SoTC values, and that increases in MSA per capita payments are not associated with increases in SoTC values. Although tobacco control policy makers view MSA payments as funds that ought to be allocated to tobacco control activities, state legislatures could divert those payments towards other uses such as funding educational needs. Such legislative decisions by each state could result from changes in relative power of political parties, changes in state fiscal conditions, or changes in public opinion with respect to tobacco control activities.

In addition, since the shares of MSA funds across states were set based on estimated tobacco-related Medicaid expenditures and the number of smokers in each state, MSA funds could provide an incentive for states to limit tobacco control related activities [2], [19]. The greater the number of smokers in each state prior to the MSA implementation (higher tobacco consumption), the higher the MSA per capita payments received by each state. In addition, later modifications to the MSA terms provided for additional funds from non-participating tobacco manufacturers; those contributions depended on the relative market share of participating and non-participating tobacco companies as well as the overall level of sales (the non-participating contributions are effectively excise taxes). This provides some incentive for states to minimize tobacco control activities in order to increase the amount of money they receive each year from the total MSA fund. This might be another reason for the negative relationship between MSA per capita payments and the SoTC values in our findings, essentially indicating that the MSA agreement may result in states being in partnership with the tobacco industry. In contrast to our findings on the level of MSA payments per capita, we find no effect from securitization of the MSA disbursements. While this practice has been controversial, our regressions cannot reject the hypothesis that states which securitize have the same average levels of tobacco control policies as states that do not. This is true both when we measure securitization as a 0/1 indicator and when we measure it as the amounts of the MSA funds been actually securitized.

The only other state characteristics that are significant predictors of tobacco control are population age and race. The coefficient of the population in the age ranges of 65 and over is negative and significant at the 5% level, implying states with older populations are more likely to have weaker overall tobacco control and fewer resources devoted to tobacco control. Moreover, racial distributions are significant predictors of the effort component of tobacco control, with states having larger minority populations also having more rigorous tobacco control.

The finding that a state with higher proportion of elderly persons is associated with lower levels of tobacco control is not surprising. Tobacco control activities are typically targeted towards young people, so states with larger proportions of older persons may not face median voter pressure to expand tobacco control. On the other hand, public health and tobacco control advocates might find it easier to develop winning political coalitions in states with large minority populations, since such subpopulations are more likely to support government activism and regulation in health and other social areas [20].

We also include a supporting information file (S1 Table) that compares each state's tobacco control spending and MSA payments received across all years of interest in the study: 2000, 2002, 2004, and 2006. As this table shows, tobacco control spending in all states across all four years were far less than the MSA payments received. Thus, although states received relatively large MSA payments, only a small portion of these payments have been allocated towards tobacco control, indicating the diversion of MSA payments to other activities.

## Discussion

Our findings suggest that MSA payments were negatively associated with overall measure of strength of tobacco control in states. State tobacco control policymaking has been evolving since the MSA was enacted and as general public opinion has become less supportive and tolerant of tobacco use; state policies toward tobacco control have generally become more aggressive. However, progress – in the sense of more openness to tobacco control by state policy makers – has not been uniform across the U.S. Maine, Nevada, Tennessee and Wyoming saw the largest improvements between 1999 and 2006 in the overall attitudes and behaviors toward tobacco control. In examining the constructs of the SoTC measure we see that they achieved these improvements largely through increases in increased funding for tobacco control programs, generally strengthened statewide coalitions, taking advantage of mass media, and substantial increases in outreach. Arizona, Florida, Massachusetts and Rhode Island fared worst. They fell behind because of reductions in funding, substantial reductions in staffing devoted to tobacco control, poor interagency relationships, and large reductions in outreach, which are again captured through constructs of the SoTC index. Another reason for the negative relationship between MSA payments and SoTC index in states could result from decisions made by state legislatures in supporting diversion of MSA funds from tobacco control activities to other non-tobacco related activities. Changes in relative power of political parties, changes in state fiscal conditions, or changes in public opinion with respect to tobacco control activities could influence decisions of state legislatures on how best to allocate MSA funds within the states.

**Figure 2:**
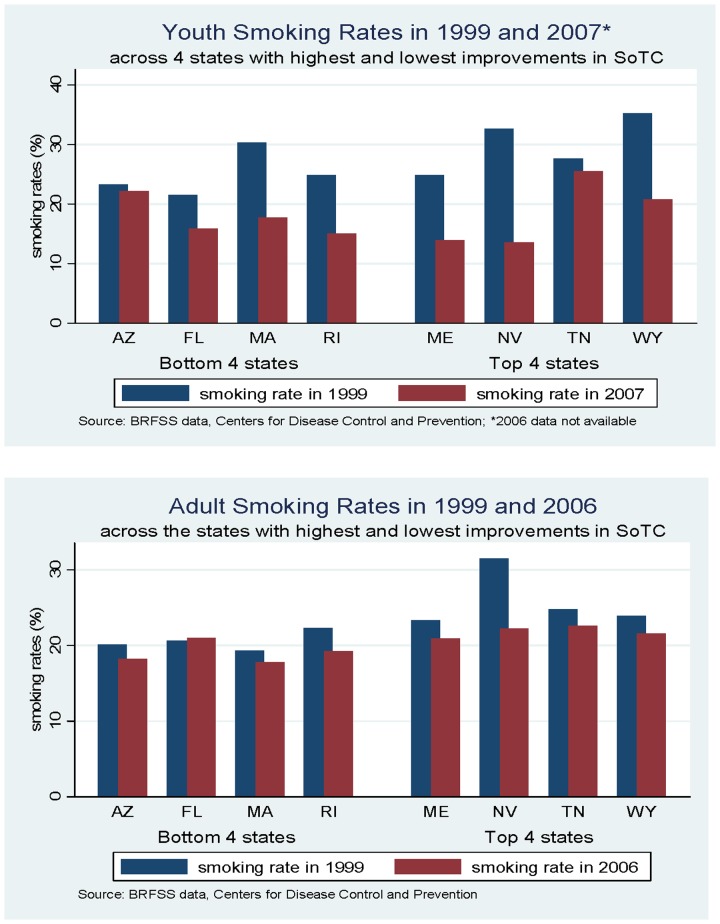
Youth and Adult Smoking Rates.

The consequences of this can be seen in the changes in smoking rates. We compiled data on youth and adult smoking rates in the states with the largest improvements in the SoTC (top 4 states) and states with the largest reduction in the SoTC (bottom 4 states) from the Centers for Disease Control and Prevention's Behavioral Risk Factor Surveillance System (Fig. 2). Generally speaking, the states with the biggest improvement began with higher smoking rates in 1999 (both youth and adult). However, they also generally saw the largest reduction in youth smoking, when compared with the states with lowest improvements. For adults, the trend is less obvious, though at least one top state (Nevada) saw substantial reductions, while one bottom state (Florida) saw a slight increase. While analysis of smoking patterns is beyond the scope of this paper, state agency attitudes seem to be associated with youth smoking rates.

### Limitations

As a validated measure of tobacco control within states, SoTC measure captures the perceptions of state agencies, local coalitions, and advocacy groups on available resources within states, their capability in using the available resources including support from leadership, and the level of effort allocated towards controlling tobacco related activities. As a result, SoTC measure may not fully capture the impact of some of the actual policy measures that states have undertaken to control tobacco activities such as regulations on advertisements of tobacco related products, and private sector funding allocated towards tobacco control. In addition, higher SoTC values are likely to lead to stronger tobacco control policies, which in turn lead to reductions in tobacco consumptions. Since MSA payments to states depends on cigarette consumption in the state, higher SoTC values could lead to reductions in MSA payments to states creating possible endogeneity bias.

Furthermore, the development of the SoTC measure does not put considerable weight on grassroots (or volunteer) efforts that has helped improve tobacco control measures in many states prior to the MSA. While such non-paid efforts, which continue to this day, have played an important role in state tobacco control efforts and contribute to the tobacco control environment in many states, these efforts may very well not be captured by the SoTC measure. Therefore, the SoTC measure included in this analysis may not be a representative of an adequate measure of actual tobacco control policy efforts that have taken place in each state and hence the results of the paper should be interpreted with caution.

## Conclusions

This is one of the first studies to investigate the relationship between MSA payments and state tobacco control environments across all states within the U.S. over time. While previous literature has examined variations across time for a single state, or variation across states for a single point in time, no study (to our knowledge) has analyzed how usage of MSA funding in all states have affected the tobacco control environment in each state. This study contributes as a first step in filling that gap in the literature.

We find that higher MSA payments are associated with weaker tobacco control environments across states. Our analysis also indicated that states with higher proportion of older individuals were less likely to have stronger tobacco control environments, while states with larger proportions of minority populations were more likely to have stronger tobacco control environments.

The results of state tobacco control environment, and the fate of MSA spending to date, can be explained largely by the realities of health policy making within states. While MSA payments received by states could be seen as funds that must be allocated towards tobacco control activities by tobacco control policy makers, state legislatures may not share the same point of view and could decide to divert those MSA funds towards other activities such as building bridges or funding educational programs. Since there was no binding agreement included in the MSA on how MSA funds should be allocated by states, legislative decisions on using MSA funds towards other activities within the state could result from changes in relative power of political parties, changes in state fiscal conditions, or changes in public opinion with respect to tobacco control activities. Given the need to balance budgets and fund basic services in the states, it is not surprising that the longer range objective of reducing tobacco use is often ignored when revenue allocation decisions are made by state legislatures.

## Supporting Information

S1 Table **Tobacco Control Spending and MSA Payments across all 4 years of interest (2000, 2002, 2004, 2006) by State.**
(DOCX)

S1 Analysis **Fixed Effect Regression Analyses.**
(DOCX)
