# Supplementary material for: Master Settlement Agreement (MSA) Spending and Tobacco Control Efforts
Source: PLoS One. 2014 Dec 15;9(12):e114706. doi: 10.1371/journal.pone.0114706 (PMC4266515; doi:10.1371/journal.pone.0114706)
Supplement: S1 Table — Tobacco Control Spending and MSA Payments across all 4 years of interest (2000, 2002, 2004, 2006) by State. (DOCX) [file pone.0114706.s001.docx]

Table S1: Tobacco Control Spending and MSA Payments across all 4 years of interest (2000, 2002, 2004, 2006) by State

|  | **2000** | | **2002** | | **2004** | | **2006** | |
| --- | --- | --- | --- | --- | --- | --- | --- | --- |
| **State** | **Tobacco Prevention Spending (Millions $)** | **MSA Payments (Millions $)** | **Tobacco Prevention Spending (Millions $)** | **MSA Payments (Millions $)** | **Tobacco Prevention Spending (Millions $)** | **MSA Payments (Millions $)** | **Tobacco Prevention Spending (Millions $)** | **MSA Payments (Millions $)** |
| Alabama | $6.00 | $163.00 | $0.60 | $114.40 | $0.36 | $100.70 | $0.33 | $94.30 |
| Alaska | $1.40 | $19.60 | $3.10 | $23.70 | $3.80 | $21.20 | $5.70 | $19.90 |
| Arizona | $29.30 | $148.90 | $36.60 | $105.40 | $23.00 | $91.60 | $23.10 | $86.00 |
| Arkansas | $0.00 | $0.00 | $16.40 | $57.40 | $18.50 | $51.50 | $17.50 | $48.30 |
| California | $88.20 | $688.60 | $134.50 | $912.70 | $90.10 | $793.50 | $79.70 | $744.50 |
| Colorado | $13.20 | $78.90 | $12.70 | $95.00 | $3.80 | $85.20 | $27.00 | $80.00 |
| Connecticut | $4.00 | $100.20 | $0.58 | $132.70 | $0.50 | $115.40 | $0.04 | $108.30 |
| DC | $0.00 | $34.90 | $0.00 | $42.10 | $0.00 | $37.70 | $0.00 | $35.40 |
| Delaware | $0.00 | $21.30 | $5.50 | $28.30 | $10.10 | $24.60 | $9.20 | $23.10 |
| Florida | $44.00 | $640.90 | $29.80 | $765.70 | $1.00 | $363.90 | $1.00 | $389.70 |
| Georgia | $15.80 | $141.20 | $20.80 | $170.00 | $12.60 | $152.60 | $3.10 | $143.20 |
| Hawaii | $9.70 | $32.50 | $4.20 | $43.00 | $8.90 | $37.40 | $5.80 | $35.10 |
| Idaho | $1.20 | $20.90 | $1.10 | $25.20 | $1.60 | $22.60 | $0.54 | $21.20 |
| Illinois | $28.60 | $267.80 | $45.90 | $322.40 | $12.00 | $289.30 | $11.00 | $271.50 |
| Indiana | $35.00 | $117.40 | $32.50 | $141.30 | $10.80 | $126.80 | $10.80 | $119.00 |
| Iowa | $9.40 | $50.00 | $9.40 | $60.20 | $5.10 | $54.10 | $5.60 | $50.70 |
| Kansas | $0.50 | $48.00 | $0.50 | $57.70 | $0.50 | $51.80 | $1.00 | $48.60 |
| Kentucky | $5.80 | $95.00 | $5.50 | $125.90 | $2.60 | $109.50 | $2.70 | $102.70 |
| Louisiana | $4.10 | $129.80 | $0.50 | $156.20 | $10.70 | $140.20 | $8.00 | $131.50 |
| Maine | $18.80 | $44.30 | $13.80 | $53.30 | $14.50 | $47.80 | $14.20 | $44.90 |
| Maryland | $30.00 | $130.00 | $20.10 | $156.60 | $14.80 | $140.50 | $9.20 | $131.80 |
| Massachusetts | $43.10 | $217.90 | $48.00 | $288.80 | $2.50 | $251.10 | $4.30 | $235.60 |
| Michigan | $0.00 | $234.80 | $0.00 | $311.20 | $0.00 | $270.50 | $0.00 | $253.80 |
| Minnesota | $35.00 | $326.70 | $28.90 | $377.90 | $20.40 | $168.50 | $22.10 | $180.80 |
| Mississippi | $31.00 | $199.50 | $20.00 | $229.00 | $20.00 | $112.50 | $20.00 | $120.50 |
| Missouri | $0.00 | $0.00 | $0.00 | $159.60 | $0.00 | $141.40 | $0.00 | $132.70 |
| Montana | $3.50 | $24.40 | $0.50 | $29.40 | $2.50 | $26.40 | $6.80 | $24.70 |
| Nebraska | $7.00 | $34.20 | $7.00 | $41.20 | $0.41 | $37.00 | $3.00 | $34.70 |
| Nevada | $3.90 | $35.10 | $4.30 | $42.30 | $4.30 | $37.90 | $4.20 | $35.60 |
| New Hampshire | $3.00 | $38.30 | $3.00 | $46.10 | $0.00 | $41.40 | $0.00 | $38.80 |
| New Jersey | $18.60 | $402.50 | $30.00 | $267.90 | $10.50 | $240.40 | $11.50 | $225.50 |
| New Mexico | $2.30 | $34.30 | $5.00 | $41.30 | $5.00 | $37.10 | $6.00 | $34.80 |
| New York | $30.00 | $688.50 | $40.00 | $912.50 | $37.00 | $793.40 | $43.40 | $744.40 |
| North Carolina | $0.00 | $125.80 | $0.00 | $166.80 | $10.90 | $145.00 | $15.00 | $136.00 |
| North Dakota | $0.00 | $21.10 | $2.50 | $25.40 | $3.00 | $22.80 | $3.10 | $21.30 |
| Ohio | $60.00 | $289.80 | $21.70 | $348.90 | $38.00 | $313.10 | $47.20 | $293.80 |
| Oklahoma | $6.30 | $59.60 | $1.70 | $71.80 | $2.50 | $64.40 | $8.90 | $60.40 |
| Oregon | $8.50 | $61.90 | $11.30 | $82.10 | $2.90 | $71.30 | $3.50 | $66.90 |
| Pennsylvania | $0.00 | $452.30 | $41.40 | $410.90 | $52.60 | $357.30 | $32.90 | $335.20 |
| Rhode Island | $2.30 | $41.40 | $3.30 | $49.80 | $2.70 | $44.70 | $2.10 | $41.90 |
| South Carolina | $1.80 | $67.70 | $1.60 | $81.50 | $0.00 | $73.10 | $0.00 | $68.60 |
| South Dakota | $1.70 | $20.10 | $2.70 | $24.20 | $0.75 | $21.70 | $0.71 | $20.40 |
| Tennessee | $0.00 | $256.40 | $0.00 | $169.10 | $0.00 | $151.70 | $0.00 | $142.40 |
| Texas | $9.00 | $839.80 | $12.50 | $1,004.50 | $7.40 | $479.90 | $7.00 | $516.10 |
| Utah | $6.00 | $25.60 | $6.00 | $30.80 | $7.20 | $27.70 | $7.20 | $25.90 |
| Vermont | $6.50 | $22.20 | $5.50 | $29.40 | $4.50 | $25.60 | $4.90 | $24.00 |
| Virginia | $13.10 | $117.60 | $19.20 | $141.60 | $17.40 | $127.10 | $12.80 | $119.30 |
| Washington | $15.00 | $118.10 | $17.50 | $142.20 | $26.20 | $127.60 | $27.20 | $119.80 |
| West Virginia | $5.90 | $51.00 | $5.90 | $61.40 | $5.90 | $55.10 | $5.90 | $51.70 |
| Wisconsin | $21.20 | $111.80 | $15.50 | $148.20 | $10.00 | $128.80 | $10.00 | $120.90 |
| Wyoming | $0.90 | $13.40 | $0.90 | $17.80 | $3.00 | $15.40 | $5.90 | $14.50 |
| Source: Campaign for Tobacco Free Kids. Note: Tobacco Prevention Spending data for 1999 was not available, thus was replaced by 2000 data. | | | | | | | | |
