# Supplementary material for: Master Settlement Agreement (MSA) Spending and Tobacco Control Efforts
Source: PLoS One. 2014 Dec 15;9(12):e114706. doi: 10.1371/journal.pone.0114706 (PMC4266515; doi:10.1371/journal.pone.0114706)
Supplement: S1 Analysis — Fixed Effect Regression Analyses. (DOCX) [file pone.0114706.s002.docx]

**Analysis S1**

*Fixed Effect Regression Analyses:*

We used a fixed effects model for the analysis. The fixed effect modeling approach allows us to control for within-individual state variation over time. The specific form of the model for the overall index is:

$e^{SoTC_{it}}= MSA_{it}\beta_{1} +X_{it}\beta_{2}+S_{i}+ \eta_{t}+\upsilon_{it}$,

where the dependent variable, exponentiated SoTC, $e^{SoTC_{it}}$is observed for individual state *i* at time *t*, the variable *MSA_it_* is a time-variant vector of regressors that includes per capita MSA disbursements, securitization indicator and securitization amount for each state at time *t*, the variable *X_it_* is a vector of other control variables (described in conceptual model) for each state at time *t*, while *S_i_* is a vector of unobserved state specific fixed effects, and *η_t_* is a vector of year indicator variables. The vectors β_1_ and β_2_ are parameters to be estimated, while *ν_it_* is a vector of error terms. Again, we used the exponentials of the dependent variable, SoTC, to better fit the data.

In addition to the model predicting overall SoTC index values, we also estimated models for the three sub-components. These models take the form:

$e^{SCI_{it}^{k}}= MSA_{it}\alpha_{1} +X_{it}\alpha_{2}+S_{i}+ \eta_{t}+\upsilon_{it}$,

where *SCI^k^_it_* represents sub-component index-*k*, the efforts score index, resource score index, and capacity score index, for each state *i* at time *t*. The vectors α_1_ and α_2_ are parameters to be estimated, while the other terms are defined as above.
